# Free-Base Carboxyphenyl Porphyrin Films Using a TiO_2_ Columnar Matrix: Characterization and Application as NO_2_ Sensors

**DOI:** 10.3390/s150511118

**Published:** 2015-05-12

**Authors:** Javier Roales, José M. Pedrosa, María G. Guillén, Tânia Lopes-Costa, Pedro Castillero, Angel Barranco, Agustín R. González-Elipe

**Affiliations:** 1 Departamento de Sistemas Físicos, Químicos y Naturales, Universidad Pablo de Olavide, Ctra. Utrera Km. 1, 41013 Sevilla, Spain; E-Mails: mariagonzalez88@gmail.com (M.G.G.); tlopcos@upo.es (T.L.-C.); pcasdur@upo.es (P.C.); 2 Instituto de Ciencia de Materiales de Sevilla, Universidad de Sevilla–CSIC, Américo Vespucio 49, 41092 Sevilla, Spain; E-Mails: angel.barranco@csic.es (A.B.); arge@icmse.csic.es (A.R.G.-E.)

**Keywords:** carboxyphenyl porphyrin, microstructured TiO_2_ film, NO_2_, optical gas sensor, thin film

## Abstract

The anchoring effect on free-base carboxyphenyl porphyrin films using TiO_2_ microstructured columns as a host matrix and its influence on NO_2_ sensing have been studied in this work. Three porphyrins have been used: 5-(4-carboxyphenyl)10,15,20-triphenyl-21*H*,23*H*-porphyrin (MCTPP); 5,10,15,20-tetrakis(4-carboxyphenyl)-21*H*,23*H*-porphyrin (*p*-TCPP); and 5,10,15,20-tetrakis(3-carboxyphenyl)-21*H*,23*H*-porphyrin (*m*-TCPP). The analysis of UV-Vis spectra of MCTPP/TiO_2_, *p*-TCPP/TiO_2_ and *m*-TCPP/TiO_2_ composite films has revealed that *m*-TCPP/TiO_2_ films are the most stable, showing less aggregation than the other porphyrins. IR spectroscopy has shown that *m*-TCPP is bound to TiO_2_ through its four carboxylic acid groups, while *p*-TCPP is anchored by only one or two of these groups. MCTPP can only be bound by one carboxylic acid. Consequently, the binding of *p*-TCPP and MCTPP to the substrate allows them to form aggregates, whereas the more fixed anchoring of *m*-TCPP reduces this effect. The exposure of MCTPP/TiO_2_, *p*-TCPP/TiO_2_ and *m*-TCPP/TiO_2_ films to NO_2_ has resulted in important changes in their UV-Vis spectra, revealing good sensing capabilities in all cases. The improved stability of films made with *m*-TCPP suggests this molecule as the best candidate among our set of porphyrins for the fabrication of NO_2_ sensors. Moreover, their concentration-dependent responses upon exposure to low concentrations of NO_2_ confirm the potential of *m*-TCPP as a NO_2_ sensor.

## 1. Introduction

Porphyrins are a family of compounds with chemical and physical properties that make them interesting for new technologies, such as solar cells [1], photodynamic therapies [2] or gas sensors [3]. In the latter field, researchers have been investigating during the last few decades the capabilities of porphyrins to detect a variety of gases. Porphyrins have been shown to be promising for the creation of sensing devices of a low price and easy use that could be applied to electronic nose technologies. In particular and based on the photophysical properties of porphyrins, their potential use for the construction of optical devices for gas sensing has attracted great attention [3,4,5,6].

Nitrogen dioxide, given its high toxicity and participation as a precursor in the formation of other contaminants, such as tropospheric ozone, fine particulate matter (PM2.5) and acid rain, is of environmental concern, especially in urban locations, where it is formed in relatively high concentrations from vehicle motor exhausts and other sources of combustion of fossil fuels. The interaction between NO_2_ and some free-base porphyrins is well documented in the literature, producing strong changes in the absorption spectrum that can be easily monitored [4,7,8,9]. Usually, porphyrin films have been made by Langmuir–Blodgett, spin coating or casting techniques, and these films have shown good sensing capabilities. Great efforts have been made to improve the sensitivity of porphyrin films to NO_2_, either by modifying the technique of deposition or combining the sensing material with host molecules, with outstanding results [10]. However, films made by these techniques are sometimes unstable given the weak interaction between porphyrin and the substrate, which leads to a short or mid-term unusability caused by porphyrin aggregation. Strong π-π interactions between porphyrins favor the formation of molecular aggregates that have been shown to be detrimental for gas sensing purposes [11].

Anchoring of porphyrin molecules to TiO_2_ by adding carboxylic acid groups to their structure has given great results in terms of film fabrication and its applicability [12]. Originally developed by solar cell researchers, the chemical binding to TiO_2_ leads to more stable films than the simple deposition onto glass. However, films made from colloidal suspensions of this material are usually thick and opaque and, hence, not appropriate for optical measurement. The elaboration of glancing angle physical deposition (GAPVD) films has attracted our attention in the last few years as their properties are nearly ideal for the construction of optical sensing devices. Although other metal oxides have been successfully used for gas sensing [13,14,15], these films feature TiO_2_ microstructured columns that make possible the covalent binding of carboxylic porphyrins to their surface, adding stability to the film properties. Moreover, they are transparent enough to be used for optical sensing and highly porous, allowing easy diffusion of gas molecules through their inner structure [16].

Besides the influence of the porphyrin-substrate interaction on the film stability, porphyrin aggregation is influenced by the molecular structure of the dye. It is known that bulky substituents can reduce aggregation by preventing porphyrin contact [8,17], and the position itself of the peripheral groups may determine different states and types of aggregation [11]. We have previously reported the effect of the position of carboxylic substituents on the anchoring to titania substrates [18], finding that metallated porphyrins bound to TiO_2_ columnar films by four points have little tendency to form aggregates and that their sensing performance towards volatile organic compounds (VOCs) is better than films made with porphyrins anchored by only one or two points. Metallated porphyrins were chosen given their good sensing properties for the detection of VOCs [5].

**Figure 1:**
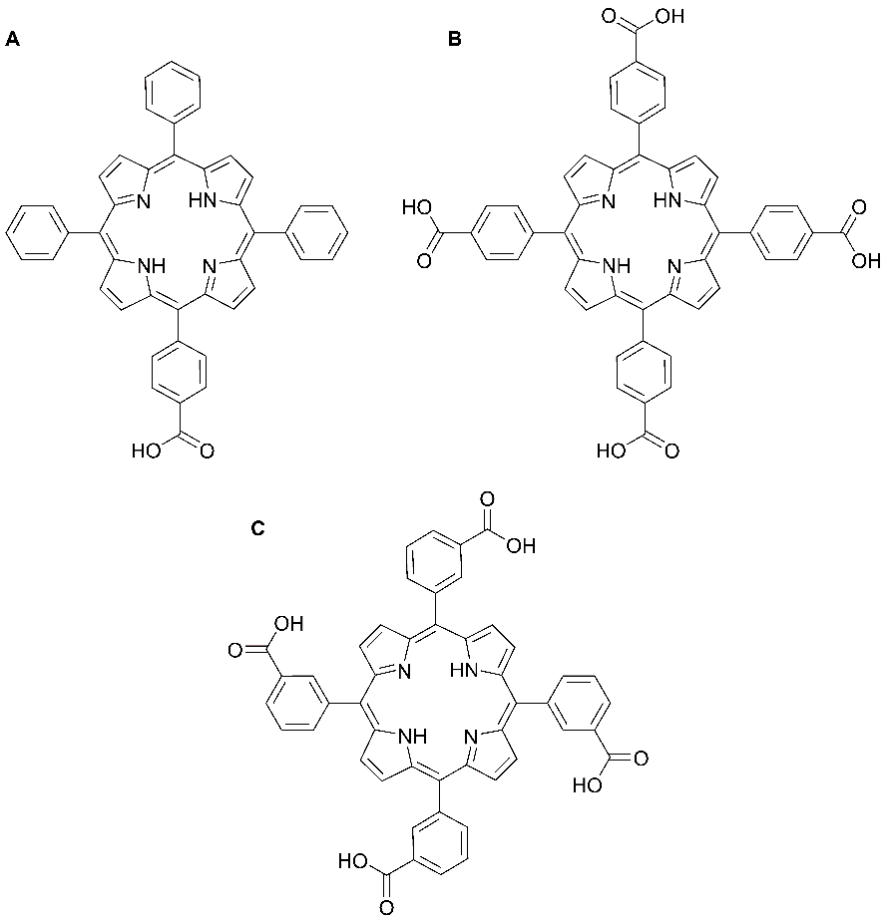
Molecular structures of: (**A**) 5-(4-carboxyphenyl)10,15,20-triphenyl-21*H*,23*H*-porphyrin (MCTPP); (**B**) 5,10,15,20-tetrakis(4-carboxyphenyl)-21*H*,23*H*-porphyrin (*p*-TCPP); and (**C**) 5,10,15,20-tetrakis(3-carboxyphenyl)-21*H*,23*H*-porphyrin (*m*-TCPP).

Here, we evaluate the anchoring effect on free-base carboxyphenyl porphyrin films using TiO_2_ microstructured columns as a host matrix and its influence on NO_2_ sensing. In this case, we have chosen unmetallated porphyrins, which are known to be good candidates for the detection of NO_2_ [4,8,11,19]. Three different free-base porphyrins have been used for this purpose: 5-(4-carboxyphenyl)10,15,20-triphenyl-21*H*,23*H*-porphyrin (MCTPP, Figure 1A), 5,10,15,20-tetrakis(4-carboxyphenyl)-21*H*,23*H*-porphyrin (*p*-TCPP, Figure 1B) and 5,10,15,20-tetrakis(3-carboxyphenyl)-21*H*,23*H*-porphyrin (*m*-TCPP, Figure 1C). The aggregation and stability of the composite films regarding the different binding geometries have been studied through UV-VIS and infrared spectroscopy. Finally, the sensing capabilities towards NO_2_ have been investigated by analyzing the responses of the composite films upon their exposure to different concentrations of the toxic gas.

## 2. Experimental Section

### 2.1. Porphyrins and Reagents

MCTPP, *p*-TCPP and *m*-TCPP were purchased from Frontier Scientific, Inc., and used as received. All reagents were purchased from Sigma-Aldrich and used without further purification.

### 2.2. Film Preparation

We prepared transparent and amorphous TiO_2_ columnar films by the GAPVD technique at an angle of deposition of 70° with respect to the evaporation source. The angle formed by the columns and the substrate was approximately 60°, with a film thickness of approximately 350 nm exhibiting an elevated porosity (total pore volume of 49%) with void apertures on the surface in the form of mesopores (pore diameter >2 nm), which also determines a relatively low refractive index value, which was found to be 1.79 [20]. Further details regarding film preparation, SEM images and structural information can be found elsewhere [16]. The high porosity of the films would allow the accessibility of porphyrins during the composite film preparation and NO_2_ during the gas sensing experiments. For specular reflectance Fourier transform infrared (FT-IR) spectroscopy, the films were deposited on gold-coated silicon substrates. Films for UV-Visible spectroscopy were prepared on glass substrates. Binding of porphyrins to the TiO_2_ films was performed by immersion of the substrates in a 5 × 10^−4^ M MeOH solution of the dye for 2 h. After this, the films were rinsed with MeOH to remove physisorbed dye molecules and dried at room temperature (~293 K) under a dry N_2_ stream.

### 2.3. Infrared and UV-Visible Spectroscopy

We studied the binding of the carboxylic porphyrins to TiO_2_ through specular reflectance FT-IR spectroscopy using a Jasco FT/IR-6200 spectrometer (Jasco Inc., Easton, PA, USA). The specular reflectance FT-IR spectra for the porphyrins were measured neat (by casting on silicon substrates) and bound to the TiO_2_ thin films.

UV-Visible spectra of the porphyrins were recorded in MeOH solution using an Ocean Optics USB4000 spectrophotometer (Ocean Optics Inc., Dunedin, FL, USA). 

### 2.4. Gas Exposure

We placed the porphyrin films in a gas testing system for the exposure to NO_2_. This system consisted of a purpose-built gas chamber with a gas inlet and an outlet, connectors for two optical fibers and a Peltier heating-cooling device (Laird Technologies, London, UK). Two Bronkhorst F-201FV mass flow controllers (Bronkhorst High-Tech BV, Ruurlo, The Netherlands) were used to control the flow rates of gases. The chamber was connected to an Ocean Optics USB4000 optical fiber spectrophotometer (Ocean Optics Inc.) to record the UV-Vis spectrum of the films during their exposure to NO_2_.

We used two gas cylinders to obtain the desired NO_2_ concentration, one containing 500 ppm NO_2_ in dry nitrogen and another containing pure dry nitrogen. Each of the cylinders was connected to a mass flow controller, and after this, both gas lines were linked together and directed into the chamber. Precise NO_2_ concentrations were obtained by modifying the flow from each of the gas cylinders and, hence, diluting 500 ppm NO_2_ with dry nitrogen.

Before NO_2_ exposure, the chamber was flushed with dry N_2_ to ensure an inert internal atmosphere, thus preventing the contamination of the samples. Then, we introduced the corresponding sample into the gas chamber while keeping a constant dry N_2_ flow through the gas inlet to allow complete desorption of possible contaminating gases that could be adsorbed on the sample. For the gas exposure phase, the gas mixture containing the desired NO_2_ concentration was introduced into the chamber until the porphyrin was fully saturated. In all cases, the samples were exposed at room temperature (~293 K). The recovery phase consisted of the introduction of dry N_2_ in the chamber while simultaneously heating the sample with the Peltier device at elevated temperature (~373 K) to desorb NO_2_ molecules from the film.

## 3. Results and Discussion

### 3.1. Composite Porphyrin/TiO_2_ Film Characterization

**Figure 2:**
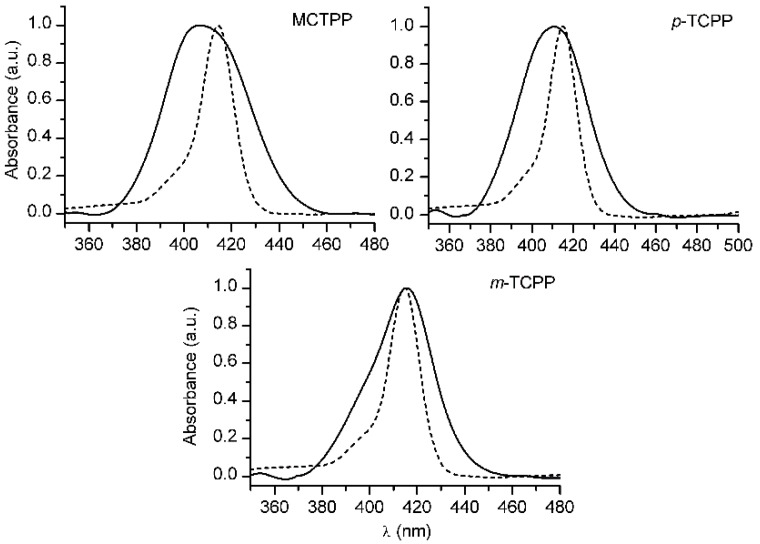
Normalized UV-Vis absorption spectra of MCTPP, *p*-TCPP and *m*-TCPP bound to TiO_2_ (solid line) and in MeOH solution (dashed line).

UV-Vis solution spectra of MCTPP, *p*-TCPP and *m*-TCPP showed their monomeric forms with Soret bands peaking at 414, 415 and 415 nm, respectively (Figure 2). Once bound to TiO_2_, each porphyrin experienced different changes in their spectra. The Soret band in the MCTPP/TiO_2_ film band was strongly broadened (full width at half-maximum: 40.5 nm) and blue-shifted (8 nm) with respect to the solution spectrum (Figure 2). Besides the broadening of the Soret band, its shape revealed the presence of, at least, two peaks. The main peak, located around 406 nm, would be generated by the formation of H aggregates of porphyrin molecules. The secondary peak, less intense, is represented by a shoulder around 417 nm and would correspond to the monomeric form of the porphyrin. When bound to TiO_2_, *p*-TCPP showed a similar behavior as MCTPP/TiO_2_ films, although slightly less broadened (full width at half-maximum: 38 nm) and blue-shifted (5 nm) (Figure 2). The shape of the Soret band also indicated the presence of more than one species in the film. However, in this case, the main peak was less blue-shifted than that of MCTPP in film, indicating a higher contribution of the monomeric form of the porphyrin to the spectrum. The spectrum of *m*-TCPP bound to TiO_2_ showed much more similarity to that obtained from its solution, with a narrower Soret band (full width at half-maximum: 30.5) and a main peak at 415.5 nm, hence practically unshifted (Figure 2). Nevertheless, a small shoulder appeared around 400 nm, corresponding to the formation of a certain number of H aggregates.

**Figure 3:**
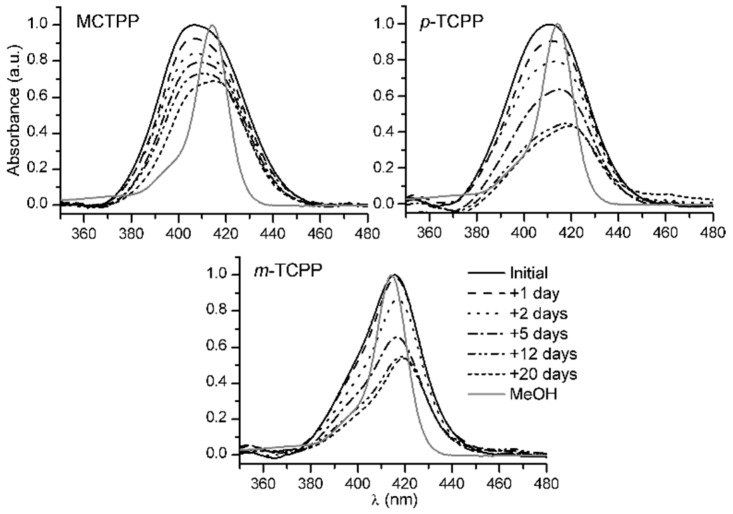
Temporal evolution of the UV-Vis absorption spectra of MCTPP/TiO_2_, *p*-TCPP/TiO_2_ and *m*-TCPP/TiO_2_ composite films over 20 days. The spectra of the porphyrins in MeOH solution are included for comparison. All spectra have been normalized with respect to their corresponding initial spectrum.

Film stability was determined by recording composite film spectra every day or every few days until 20 days from the preparation of the films (Figure 3). During this time, except during their UV-VIS spectra measurement, samples were stored in the dark and preserved from contact with any contaminating gases. The effect of time on all porphyrin films had two main results. First, all films featured a loss of intensity in their absorbance, which can be attributed to the formation of large porphyrin clusters. Second, there was a decrease in the contribution of the lower wavelength peak to the film spectrum in all three cases, probably due to the lower stability of H aggregates, which led to a progressive red shift of the Soret band over time. In MCTPP and *p*-TCPP films, the shape of the spectra after 20 days showed a change in the position of the main peak to a wavelength of 415.5 nm and 419 nm, respectively. The spectrum of *m*-TCPP after 20 days was modified as explained; however, the main peak was still primarily composed of the monomeric species of the porphyrin as in the initial spectrum, and the overall change observed in the film was smaller than in the cases of MCTPP and *p*-TCPP films.

**Figure 4:**
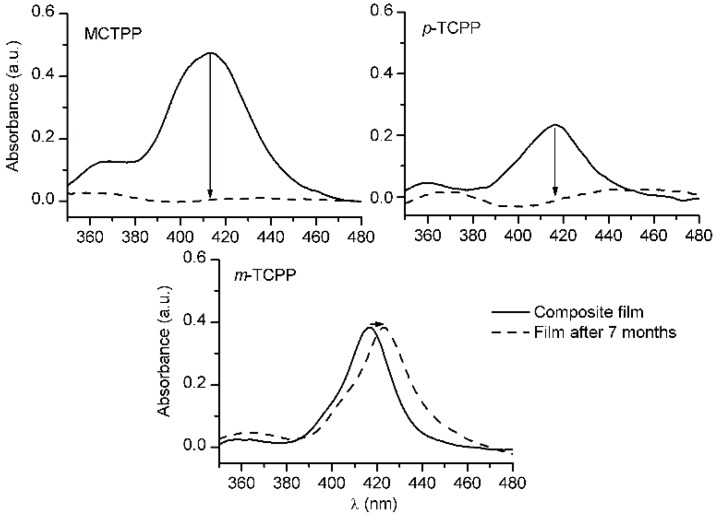
UV-Vis absorption spectra of MCTPP/TiO_2_, *p*-TCPP/TiO_2_ and *m*-TCPP/TiO_2_ composite films after preparation (solid line) and seven months later (dashed line).

Further information about film stability over time was obtained through the analysis of the spectral changes experienced by the composite films after a long period of time (Figure 4). Seven months after their preparation, MCTPP and *p*-TCPP film spectra showed a flat line without any trace of their respective Soret bands. Decomposition of the molecules or evaporation of the porphyrins from the surface could explain this bleaching if all films had shown a similar behavior. However, the spectra of *m*-TCPP films after seven months were similar to those obtained initially, showing no loss in absorbance. Taking into account the higher stability and less aggregation already observed in *m*-TCPP films, the bleaching experienced by MCTPP and *p*-TCPP films may be attributed to the extended formation of porphyrin clusters, leading to an uncolored film.

The differences in the aggregation state and stability of the porphyrins in film can be explained through their different binding to the TiO_2_ matrix. MCTPP would be anchored by its only carboxylic group, which leads to a flexible union between porphyrin and TiO_2_ that would allow dye molecules to tilt and rotate, facilitating contact between porphyrin rings and, thus, their aggregation. In the case of *p*-TCPP, its four carboxylic groups are located in the *para* position of the phenyl groups, leading to a planar structure. With this arrangement, the binding through four points is almost impossible, the presence of one or two anchoring points being more plausible. Therefore, *p*-TCPP would also be bound with its tetrapyrrolic ring normal to the TiO_2_ surface, allowing face to face interaction and subsequent molecular aggregation. The structure of *m*-TCPP is similar to *p*-TCPP, but in this case, the four carboxylic groups are situated in the *meta* position of the phenyl groups, hence orientated perpendicular to the plane of the molecule. This configuration allows the porphyrin to anchor by four points to the TiO_2_ matrix while lying flat on the surface, resulting in a fixed position for each porphyrin that hinders aggregation and provides higher stability to the film than in the case of MCTPP or *p*-TCPP.

**Figure 5:**
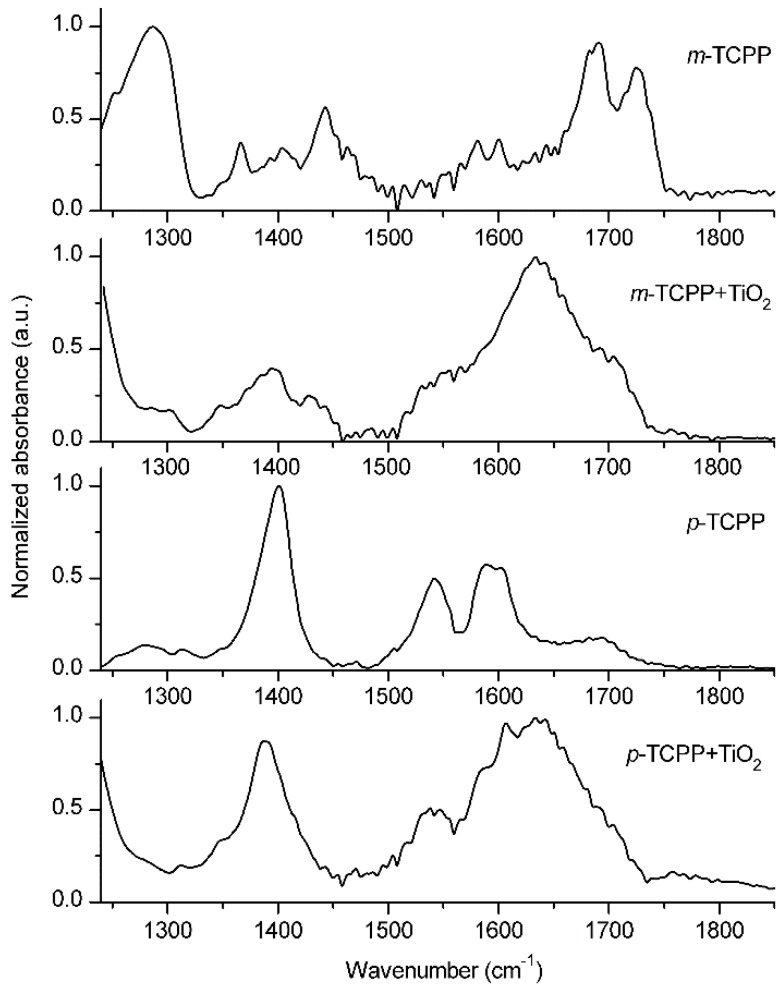
Specular reflectance FT-IR spectra of *m*-TCPP and *p*-TCPP neat (by casting on silicon substrates) and bound to TiO_2_.

Further confirmation regarding binding modes of porphyrin molecules to TiO_2_ and their influence on aggregation was obtained by the analysis of their IR spectra. Specular reflectance FT-IR spectra of *m*-TCPP and *p*-TCPP porphyrins neat and bound to TiO_2_ are shown in Figure 5. The binding interaction between the porphyrin and the metal oxide surface is revealed by the comparison of changes in the region of the carbonyl group in the FT-IR spectra. Neat samples of *m*-TCPP showed strong bands at 1725 cm^−1^ and 1286 cm^−1^, which are characteristic of the ν (C=O) stretch and the ν (C-O) stretch of the carboxylic acid groups, respectively. In the case of *p*-TCPP, where the -COOH groups are situated in the plane of the tetrapyrrole macrocycle, the extensive hydrogen bonding of the carboxylic acid groups resulted in a shift to lower frequency of the ν (C=O) stretch at 1595 cm^−1^ and a shift to higher frequency of the ν (C-O) stretch at 1400 cm^−1^ [21].

**Figure A1:**
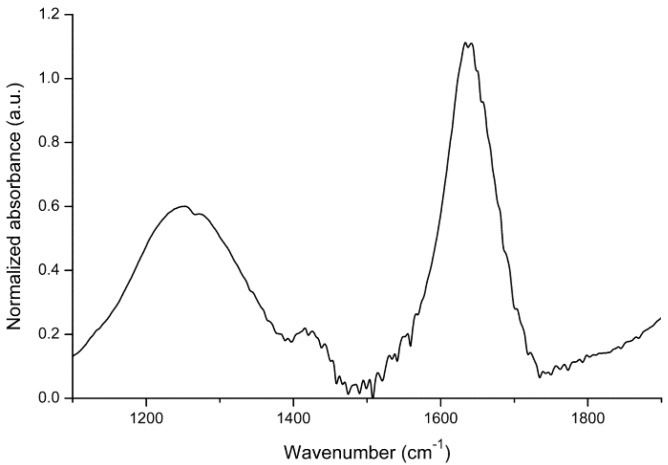
Specular reflectance FT-IR spectrum of a columnar TiO_2_ film [18]. With permission from [18].

Binding of *m*-TCPP to TiO_2_, resulted in the disappearance of the ν (C=O) and ν (C–O) stretching modes and the appearance of new bands in the 1395–1430 cm^−1^ and 1530–1560 cm^−1^ regions, corresponding to the symmetric and asymmetric ν (CO_2_^−^) stretches, respectively. A strong and broad band around 1635 cm^−1^ generated by the free TiO_2_ molecules of the columnar film hindered partially the bands corresponding to the asymmetric ν (CO_2_^−^) stretch, but these were still evident. In the case of *p*-TCPP/TiO_2_, the bands generated by the C=O and C-O stretching modes were still partially present with a slight broadening of the latter. In this case, the appearance of the band corresponding to the symmetric ν (CO_2_^−^) stretch is not so evident due to overlapping with the remaining ν (C-O) stretch band, although its broadening indicates the contribution of the former. Moreover, the changes in the 1500–1750 cm^−1^ region, where the asymmetric ν (CO_2_^−^) stretch band was expected to appear, were hindered by the presence of the band corresponding to the free TiO_2_ molecules (Figure A1 in the Appendix).

Chemical binding of carboxylic acids to TiO_2_ colloidal films has been associated with the disappearance of the bands corresponding to the ν (C=O) and ν (C-O) stretching modes and with the appearance of strong and broad bands at ~1400 cm^−1^ and ~1550 cm^−1^, characteristic of the symmetric and asymmetric ν (CO_2_^−^) stretches, respectively [12]. These spectral changes have been found to be compatible with chelating and/or bidentate binding modes of the carboxylate groups on the TiO_2_ surface [12,22,23,24,25].

The analysis of the IR spectrum of *m*-TCPP/TiO_2_ revealed that this was consistent with the absence of free carboxylic acid groups, given that C=O and C-O stretching modes disappeared completely. This suggests a planar situation of the porphyrin macrocycle with respect to the titania surface in which all carboxyl groups are bound to the TiO_2_ [12]. However, in the case of *p*-TCPP/TiO_2_, the stretching modes corresponding to C=O and C-O disappeared only partially, indicating the presence of free carboxylic acid groups coexisting with carboxylate groups bound to TiO_2_. As a result of this, and due to the planar structure of the *para* substituted *p*-TCPPs, it can be expected that they are bound only by one or two of its four carboxyl groups to the metal oxide surface, resulting in a perpendicular orientation of the molecule with respect to the surface that allows them to interact (face to face) with other molecules, causing aggregation [12,26].

From these results, we can infer that MCTPP will be bound to the TiO_2_ through the same binding modes as *p*-TCPP. Although studying its IR spectrum was not possible, the molecular structure of MCTPP, with only one carboxyl group, can only be bound to TiO_2_ by one anchoring point. Hence, and due to its planar structure, MCTPP molecules are likely to be perpendicularly oriented with respect to the surface, which allows the formation of face to face aggregates.

### 3.2. NO_2_ Detection

**Figure 6:**
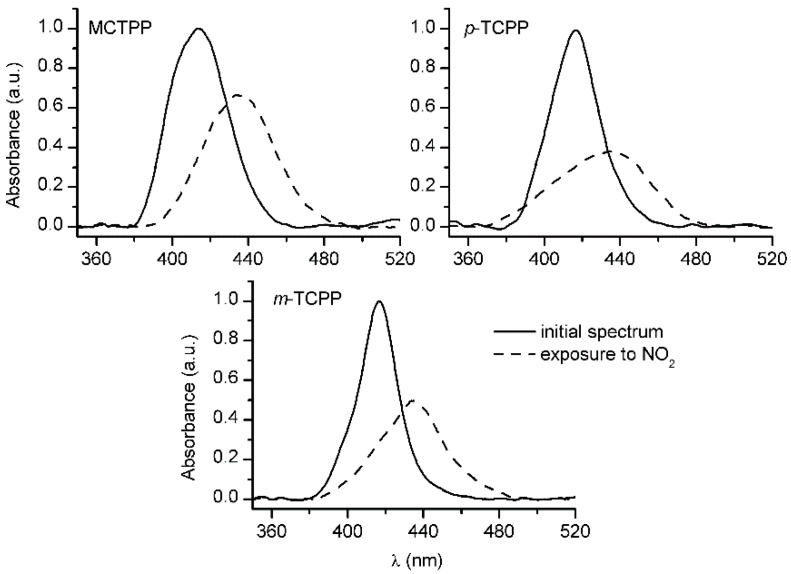
Pre-exposure (solid line) and exposure (dashed line) spectra of MCTPP/TiO_2_, *p*-TCPP/TiO_2_ and *m*-TCPP/TiO_2_ composite films upon exposure to 500 ppm NO_2_.

MCTPP/TiO_2_, *p*-TCPP/TiO_2_ and *m*-TCPP/TiO_2_ composite films were exposed to 500 ppm NO_2_ to assess their sensing performance. The exposure of the films to the toxic gas resulted in the disappearance of the typical Soret band of the porphyrins and the appearance of a new band around 435 nm (Figure 6), which is consistent with results from other authors using different free-base porphyrins [4,8,11,27]. These spectral changes are well documented, and given that porphyrins are electron-rich systems and NO_2_ is a strong oxidizing agent, a charge transfer process in the form of oxidation is the most probable mechanism for such behavior [4,8,11,17]. The magnitude of the spectral changes was high in all cases, but recovery of the samples was only partially achieved after heating the samples under a N_2_ stream, which makes them good candidates for single-use NO_2_ sensors. Other recovery strategies are a subject of further research.

**Figure 7:**
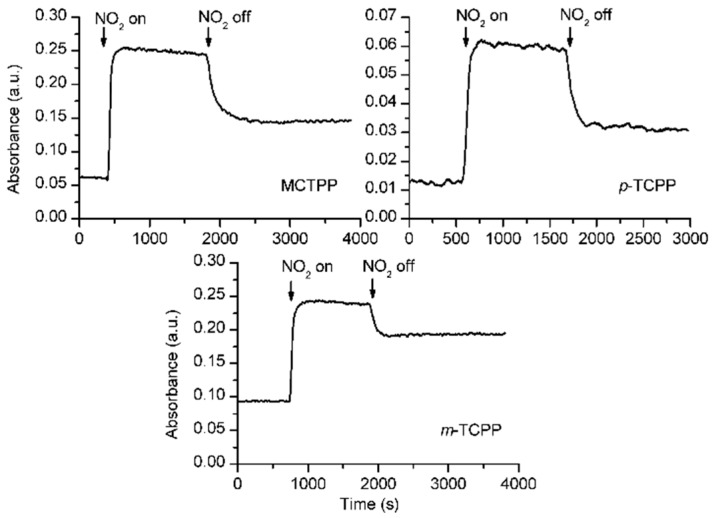
Kinetics of the exposure of MCTPP/TiO_2_, *p*-TCPP/TiO_2_ and *m*-TCPP/TiO_2_ composite films to 500 ppm NO_2_ measured at 447, 445 and 442 nm, respectively.

The speed of response was analyzed by monitoring the absorbance at one of the wavelengths of maximum change during the exposure of the composite films to 500 ppm NO_2_ (Figure 7). All porphyrins featured fast changes with similar response shapes. After the signal was stabilized, we proceeded to the recovery phase with N_2_ while heating the samples, which led to a partial reversibility, as indicated before. To quantify the speed of response, we calculated t_50_, which is the time taken for the absorbance to reach 50% of its maximum value. The obtained t_50_ values for MCTPP/TiO_2_, *p*-TCPP/TiO_2_ and *m*-TCPP/TiO_2_ composite films were 31, 30 and 33 s, respectively, confirming the fast response of all films.

The information obtained from the analysis of the spectral changes and the speed of response indicated that films made with MCTPP, *p*-TCPP and *m*-TCPP worked similarly upon their exposure to NO_2_. Therefore, there is no clear evidence that the different anchoring geometries of our porphyrins have a strong influence on the sensing capabilities of the composite films. However, the less aggregated state and much better stability over time achieved by *m*-TCPP once bound to TiO_2_ suggests this as the best candidate among our set of porphyrins to be used as a NO_2_ sensor. Hence, from this point on, we will analyze the spectral responses of *m*-TCPP/TiO_2_ composite films towards low concentrations of NO_2_ to further characterize their sensing performance.

**Figure 8:**
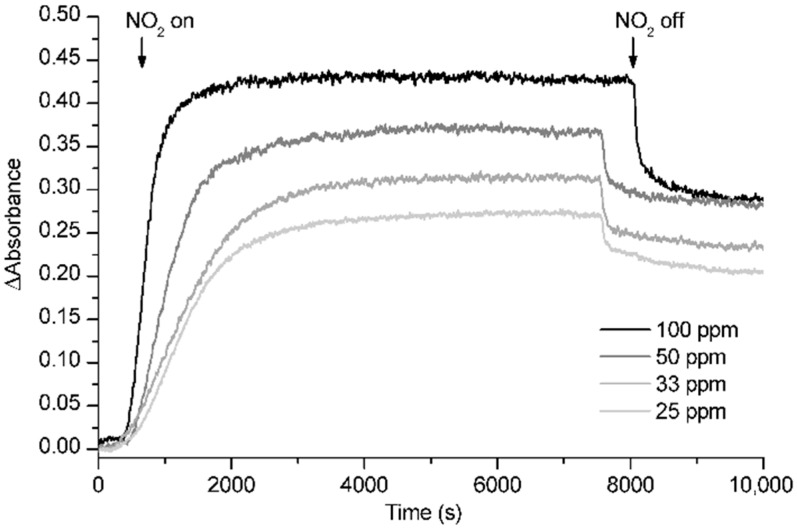
Kinetics of the exposure of *m*-TCPP/TiO_2_ composite films to 25, 33, 50 and 100 ppm NO_2_ at a wavelength of 442 nm.

In order to study the concentration dependence of the response of *m*-TCPP/TiO_2_ composite films to NO_2_, we exposed the samples to 25, 33, 50 and 100 ppm NO_2_, obtained by diluting a gas stream containing 500 ppm NO_2_ with another N_2_ stream. The exposure to increasing concentrations of NO_2_ led to increasing changes in the films’ spectra, indicating a concentration-dependent behavior of the system (Figure 8). The values of the magnitude of response, measured as the increment of absorbance at the maximum change wavelength and corresponding to 25, 33, 50 and 100 ppm NO_2_, were 0.273, 0.315, 0.372 and 0.433, respectively, confirming that the response was intensified as the concentration of NO_2_ was increased. We also calculated t_50_ to account for different speeds of response after the exposure to each of the gas concentrations. The obtained t_50_ values for the exposure of the composite films to 25, 33, 50 and 100 ppm NO_2_ were 1100, 1040, 620 and 420 s, respectively. These values confirm that the response, although slow at low concentrations, becomes faster when more NO_2_ molecules are available, further confirming a concentration-dependent behavior of *m*-TCPP/TiO_2_ composite films. These results suggest that the system is appropriate to be used for quantification purposes through the performance of a simple calibration for the desired range of concentrations.

Further characterization of the concentration dependence was obtained by applying an isotherm model. The Langmuir adsorption isotherm [28] can be used for the study of the adsorption of different types of molecules onto different materials [20,29,30,31] and, in our case, is a helpful tool to describe the adsorption/desorption equilibrium of NO_2_ molecules onto the porphyrin film solid surface. The model is usually expressed by:

(1) $$\frac{n_{ads}}{N_{S}}=\frac{\text{λ}c}{1+\text{λ}c}$$

where *n_ads_* is the number of gas molecules adsorbed (which is proportional to the change in Soret band absorbance, *ΔAbsorbance*), *N_s_* is the number of adsorption sites, λ is a constant relating to the adsorbability of the gas and *c* is the concentration of the gas, [NO_2_]. Rearrangement of Equation (1) leads to the linear form of the Langmuir adsorption isotherm:

(2) $$\left(\frac{c}{n_{ads}}\right)=\left(\frac{c}{N_{S}}\right)+\left(\frac{1}{N_{S}\lambda }\right)$$

Therefore, the plot of *c/n_ads_ versus* *c* should generate a straight line if the data points follow the Langmuir model.

**Figure 9:**
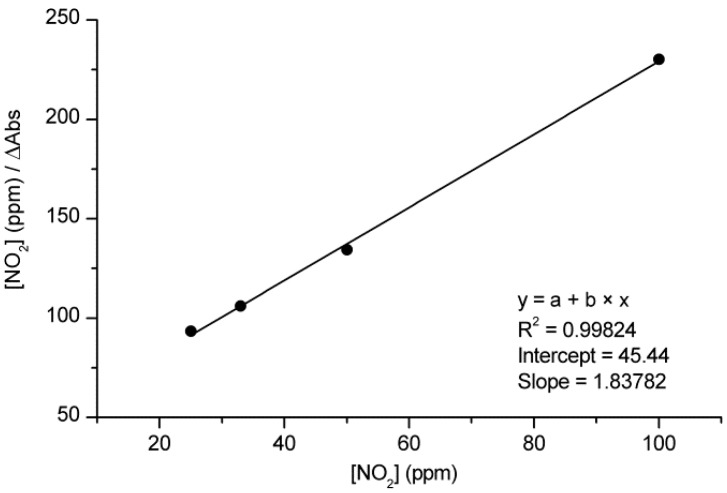
Langmuir adsorption plot for *m*-TCPP/TiO_2_ composite films upon exposure to 25, 33, 50 and 100 ppm NO_2_.

In our case, the plot of *c/ΔAbsorbance vs.* *c* fitted a straight line (Figure 9), indicating that the conditions for the Langmuir adsorption model are satisfied. Therefore, and according to the assumptions of this model, it can be concluded that the activation energy of adsorption is the same for all binding sites in the porphyrin film, that there are a fixed number of localized surface sites present on the surface and that NO_2_ molecules striking a surface site that is already occupied do not adsorb onto that particular site.

## 4. Conclusions

Composite films based on microstructured columnar TiO_2_ films and free-base porphyrins have been prepared in order to analyze their gas-sensing capabilities towards NO_2_. These films, owing to their ease of preparation and high stability, are a convenient alternative to those obtained by more complicated and time-consuming techniques, such as Langmuir–Blodgett, which often produce films of unsatisfactory stability.

UV-VIS spectra of MCTPP/TiO_2_, *p*-TCPP/TiO_2_ and *m*-TCPP/TiO_2_ composite films have revealed that *m*-TCPP/TiO_2_ films are the most stable, showing less aggregation than the other porphyrins and peaking at the same wavelength as its monomeric solution.

IR spectroscopy has shown that *m*-TCPP is bound to TiO_2_ through its four carboxylic acid groups, while *p*-TCPP is anchored by only one or two of these groups. MCTPP, given its structure, can only be bound to the TiO_2_ by one carboxylic acid. As a result of this, *p*-TCPP and MCTPP flexible binding allows them to tilt and rotate, producing aggregates. This effect is greatly reduced by the more fixed anchoring of *m*-TCPP, which enhances the stability of its films.

The exposure of MCTPP/TiO_2_, *p*-TCPP/TiO_2_ and *m*-TCPP/TiO_2_ composite films to NO_2_ has resulted in important changes in their UV-VIS spectra, revealing good sensing capabilities that were similar in all cases, with fast and intense responses. Given that no important differences have been found in the responses of the different porphyrins upon their exposure to NO_2_, *m*-TCPP would be the best candidate for the fabrication of NO_2_ sensors, given its higher stability.

Finally, the exposure of *m*-TCPP/TiO_2_ composite films to low concentrations of NO_2_ has shown concentration-dependent responses, increasing their magnitude and speed of response as the concentration of the gas increased, hence confirming the potential of *m*-TCPP as a NO_2_ sensor.
